# Milk and Milk-Contracts

**Published:** 1913-05-03

**Authors:** Everitt E. Norton

**Affiliations:** Medical Superintendent of the Isleworth Infirmary.


### MILK AND MILK-CONTRACTS.

By EVERITT E. NORTON, M.D., D.P.H., etc., Medical Superintendent of the Isleworth Infirmary.

### I.—Institution Milk Supplies.

The milk of the cow has acquired a dietetic position unique in importance and quite exceptional in character. Its value as a food, its peculiar liability to contamination and to rapid deterioration, and the difficulties connected with town supplies have raised questions which are of vital importance to the public health. Apart from the use of this milk as a food for the healthy, and as an easily assimilable form of fluid nourishment for the sick adult, a steadily and widely increasing dependence is being placed upon it as the whole source of nutrition during infancy. Whether this practice in infant-feeding is due in a majority of instances to the disinclination or to the inability of mothers to feed their infants at the breast is immaterial; the importance of the condition and quality of the substituted food is unquestionable, and grave doubts and differences of opinion exist as to the relative value of the methods in vogue in connection with its preparation. It is only comparatively recently that the importance of the control in detail of urban milk supplies has even begun to be adequately recognised. The public in this country has taken, so far, but a languid interest in the matter; it unquestioningly and trustingly accepts the conditions of its milk supply as the business of the health authorities.

There can be no doubt that many changes have taken place in the methods of the farmer and the dairyman; though it is difficult to find out the precise details of these changes, to what extent they have been adopted, or in what degree they are of benefit to the consumer. Dr. Ralph Vincent says that "recent developments on the part of milk vendors have been retrogressive. It is now becoming a comparatively common practice to *pasteurise* milk, so that the consumer does not obtain the natural article, but a cooked milk. With a perverse ingenuity, refrigerating machinery is employed to remove the heat from the milk so pasteurised, instead of being employed to refrigerate the milk as it comes from the cow. It cannot be too strongly insisted upon that a milk vendor who pasteurises his milk pronounces his own condemnation . . . ."

The terms of trade contracts are not readily made known; they differ but little, however, from those of the contracts which are entered into for the supply of large public institutions for the sick. It seems, therefore, that it may be useful to consider what is being secured under the terms of these latter contracts, not only because of the information so afforded concerning the present conditions of London milk supplies in general, but with a view to the possibility, in the light of modern scientific research, of their modification or improvement.

With these objects in view I have made inquiries concerning the contracts under which some 19,250 hospital and infirmary patients, including a considerable percentage of young children and infants, are supplied with milk, the quantity consumed being upwards of 25,000 imperial gallons weekly.

The terms of the contracts under which milk is at present supplied to large London institutions vary, within limits widely differing, from brief definitions to specifications which, if adhered to, should ensure the delivery of a quality of milk as good as is obtainable in London under existing conditions.

Contracts usually commence by defining in general terms the milk to be supplied as "best," "new," "genuine," "pure," "fresh," "good," or "country"; such expressions as "clean," "not condensed," or "guaranteed" being occasionally used.

A minimum standard is almost invariably laid down as to the content of total solids, milk-fat, and solids not fat; very rarely it is stated that the specific gravity is to be "not less than 1030."

The adopted minimum for total solids is often 12.5 per cent., but is sometimes as low as 11.5 per cent.; that for solids not fat is frequently 8.5 per cent., but is sometimes 9 per cent.

With regard to the cream-content the milk is usually required to be "unskimmed," "not skimmed," "as milked from the cow," "unseparated," "not separated," or "whole milk with all its cream"; or the milk is required to have "no cream removed," to contain "all the natural butter-fat," or "all the cream as yielded by the healthy cow fully milked out."

The minimum content specified for milk-fat varies from 3 per cent. to 3.5 per cent., and is often 3.3 per cent. Not infrequently the standard adopted varies, according to the time of year, between 3 per cent., 3.25 per cent., and 3.5 per cent., being then, of course, higher during certain of the winter months. Occasionally it was laid down that any churn delivered is to show a yield of not less than 3 per cent., or an average yield is required to be given by the morning and afternoon deliveries.

In a few instances the standard adopted depends upon the measured quantity of cream which separates on standing, instead of the estimated percentage weight, and is defined at 10 per cent., or not less than 10 per cent., on standing eight hours.

It is remarkable that, whilst it is often found convenient to lay down limits of certain hours before which deliveries are to be made, provision is very rarely made concerning what may be termed the age of the milk at the time of delivery. It is, moreover, only in somewhat rare instances that the milk is ordered to be "raw," "untreated," or "untreated by heat."

Many contracts, however, contain a clause, either stating that the milk is to be "unadulterated" or forbidding the addition of certain specified substances; the substances named are "added water," "chemical preservatives," "any preservative," "antiseptics," "drugs," "chemicals of any kind added for preservative or other purposes," "sugar," "boric acid," "salicylic acid," and "formaldehyde and its compounds or preparations of it." Further occasional provisions are that no "foreign substances," "colouring matter," or "foreign colouring matter" must be present, and that the milk "must be free from disagreeable odours and tastes," or from "any offensive taste such as might arise from unsuitable feeding or other cause."

In some contracts, otherwise fairly typical, unusual provisions appear; one, curious and interesting, is to the effect that the milk is to be exchanged "if sour or if it will not boil without curdling." Again, there are well-intentioned attempts to improve the position which often, through timidity, fall short of their object; instances of such provisions are requirements that the place of origin of the milk shall be stated or that the churns shall have the name of the farm engraved upon them, vague demands as to cleanliness at farms over the management of which the contractor probably has no control, and a clause that the milk "*should*" be cooled before delivery.

Hospital Contracts.

In a separate class are certain contracts, for hospital and other supplies, the terms of which, whilst more definite and binding, are yet such as can fairly be expected to be fulfilled by the trade. These stipulations resemble those which I shall myself suggest; they are in many ways satisfactory, and aim at a supervision of the conditions under which the milk purchased is produced and delivered.

In such specifications reference is made, in addition to many of the points above-mentioned, to: (i) The health of cows; (ii) straining and, in some instances, cooling of the milk; (iii) the construction and cleanliness of railway churns, cans, etc.; (iv) the cleanliness of the milkers; and (v) the inspection of farms, cows, etc. It is further laid down that certain information and facilities for inspection are to be afforded, and that the milk is not to be "pasteurised" before delivery.

I am intentionally omitting any mention of such general terms and conditions as are applicable to all forms of contract, which are not within the scope of my inquiry.

